# Dietary behavior and knowledge of dental erosion among Chinese adults

**DOI:** 10.1186/1472-6831-10-13

**Published:** 2010-06-03

**Authors:** CH Chu, Karie KL Pang, Edward CM Lo

**Affiliations:** 1 Faculty of Dentistry, The University of Hong Kong, Hong Kong, China; 2 Public Opinion Program, Social Science Research Center, The University of Hong Kong, Hong Kong, China

## Abstract

**Objectives:**

To study the dietary behavior and knowledge about dental erosion and self-reported symptoms that can be related to dental erosion among Chinese adults in Hong Kong.

**Methods:**

Chinese adults aged 25-45 years were randomly selected from a list of registered telephone numbers generated by computer. A telephone survey was administered to obtain information on demographic characteristics, dietary habits, dental visits, and knowledge of and presence of self-reported symptoms that can be related to dental erosion.

**Results:**

A total of 520 participants were interviewed (response rate, 75%; sampling error, ± 4.4%) and their mean age was 37. Most respondents (79%) had ever had caries, and about two thirds (64%) attended dental check-ups at least once a year. Respondents had a mean of 5.4 meals per day and 36% had at least 6 meals per day. Fruit (89%) and lemon tea/water (41%) were the most commonly consumed acidic food and beverage. When asked if they ever noticed changes in their teeth, most respondents (92%) said they had experienced change that can be related to erosion. However, many (71%) had never heard about dental erosion and 53% mixed up dental erosion with dental caries.

**Conclusion:**

Hong Kong Chinese adults have frequent intake of food and many have experienced symptoms that can be related to dental erosion. Their level of awareness of and knowledge about dental erosion is generally low, despite most of them have regular dental check-ups. Dental health education is essential to help the public understand dental erosion and its damaging effects.

## Background

Dental erosion is defined as tooth surface loss caused by chemical or electrochemical processes of non-bacterial origin [1]. It usually involves acid from endogenous (eg gastric) and exogenous (eg dietary) sources (Table 1). Acid erosion, or corrosion, of the tooth surface is now recognized as a major cause of tooth wear [2]. Unless halted, enamel erosion often leads to widespread exposure of dentin, resulting in an unsightly appearance of teeth, tooth hypersensitivity, and consequently reduced chewing function. Advanced cases may also involve the destruction of dentin and pulp. Hence, treatment may be complicated, difficult, and challenging. The costs of treatment and rehabilitation are also often very high. Because the world's population is aging with more elderly people and the advance in preventive dental care allows more and more people to retain their teeth into old age [2], the diagnosis and treatment of acid erosion, as well as other forms of tooth wear including attrition (wear against other teeth) and abrasion (wear against other surfaces), will become an essential and important part of the daily practice of dentists. Public education will have a potentially useful role to play in the prevention of dental erosion and tooth wear and in prolonging the longevity of teeth.

**Table 1:** Primary causes of intrinsic and extrinsic dental erosion *.

**Intrinsic causes**
1. Anatomical and neuromuscular defects such as hiatal hernia, esophageal diverticulosis, obstructions, gastroesophageal reflux disease (GERD)
2. Psychological problems such as bulimia nervosa, anorexia nervosa, alcoholism, stress rumination
3. Irritation of the gastric mucosa from spices, acidic foods and drinks, alcohol, aspirin and other drugs, infections
4. Medical conditions such as uremia, ascites, diabetes, peptic ulcer, morning sickness during pregnancy
**Extrinsic causes**
1. Diets, such as acidic citrus and other fruits, fruit juices, carbonated acidic beverages and sports drinks/waters, beers and herbal teas, vinegars and pickled conserves
2. Medications, such as non-encapsulated hydrochloric acid replacement, chewing vitamin C tablets, iron tonics, amino acid supplements, salivary stimulants
3. Occupations, such as wine tasting or jobs that involve working around acidic industrial vapors
4. Sports, such as swimming in improperly gas-chlorinated pools ('swimmer's teeth')

* Source: Smales and Kaidonis, 2006

Before appropriate public education programs can be designed, prevalence data are needed to understand the scope of the problem for different countries, different age groups, and different conditions or diets. Only limited longitudinal data are currently available in the literature. For example, among Norwegian adults, the prevalence of dental erosion was shown to increase from 8% in 1990 to 15% in 1999 [3]. Most other data are cross-sectional and show a wide range of prevalence rates. For example, in one clinical survey, 7% of 126 adult Nigerians had dental erosion [4] and in a controlled study of Finnish adults in the Metropolitan Helsinki area, the prevalence was 5% [5]. By contrast, a much higher prevalence of 46% was found among 179 young adults in Beijing, China [6]. In a review, Smales and Kaidonis [2] concluded that tooth erosion may affect about 35% of permanent teeth of adolescents worldwide. Data on advanced erosion are also scant and variable. Dentin erosion was found in 8% of young adults and 13% of middle-aged adults in a Swiss study [7]. In addition, 28% of maxillary anterior teeth among a sample of Saudi young men showed pronounced dental erosion that might involve the pulp [8].

The development of dental erosion has been shown to be correlated with the consumption of cola-type drinks and fruit juices [6,9]. In the UK, there is growing evidence that a major cause of tooth erosion is gastroesophageal reflux [10]. A large proportion of people with anorexia and bulimia nervosa develop dental erosion from repeated vomiting [11]. In addition, people with chronic alcoholism, [12] wine tasters [13], wine makers [14], and people exposed to acid fumes in their line of work [15] have all been reported to have an increased risk of tooth erosion. Although acid erosion may not yet be a serious dental heath problem in southern Chinese preschool children [16], there are no published studies to date on the prevalence of dental erosion in Chinese adults. Since a pilot study is desirable before conducting a clinical survey on dental erosion, this study thus investigated the dietary behavior of a Chinese adult population as well as their knowledge about and prevalence of signs and symptoms that can be related to dental erosion.

## Methods

A questionnaire survey was conducted by telephone interviews in February, 2008. The target population was Chinese adults aged 25 to 45 years living in various districts in Hong Kong. The questionnaire was developed and tested by the authors after a focus group discussion held among lay adults and dentists to identify important issues. The questionnaire composed of four sections. The first section asked for demographic data (age, sex and educational level). The second comprised questions that aimed to assess dental visit behavior, caries experience, and dietary habits. The third section contained questions to measure respondents' knowledge and experience of signs and symptoms that can be related to dental erosion including (1) teeth turning yellow; (2) teeth become thinner, (3) slight twinge upon consumption of hot, cold, sour or sweet food, (4) cracks on the edge of teeth; and (5) shiny tooth surface. Respondents who had three or more of the five signs and symptoms would be considered people at risk to dental erosion. The final section asked for intended course of action if they had dental erosion. The questionnaire (Additional file 1) was reviewed by a specialist in dental erosion who was not involved in this study for face and content validity. The full questionnaire and details of survey method can be found in the HKU POP website in the Research Report Section http://hkupop.hku.hk/: Survey on Hong Kong People's Dietary Behaviour in Relations to Acid Erosion http://hkupop.hku.hk/english/report/dental/resources/questionnaire.pdf.

Forty trained field workers who were experienced in conducting telephone surveys were provided by the Public Opinion Program (POP), Social Science Research Center, The University of Hong Kong, to administer the questionnaire in Chinese. A representative cohort of Hong Kong Chinese residents was chosen by electronic random sampling methods routinely used by the POP for all their telephone surveys. A written version of the telephone survey was given to each interviewer, who adhered strictly to the structure and format of the script provided. After self-introduction by the interviewer, an initial screening question was asked to check if the respondent was between 25 and 45 years of year and could speak in Chinese. If the age given was not within this range, the respondent was asked to invite a Chinese speaking person aged 25 to 45 years to answer the telephone. If no one in the household could satisfy the inclusion criteria, the interviewer terminated the call. All calls were kept anonymous and respondents were informed of the study protocol before giving consent. The study was approved by the Social Science Research Center, The University of Hong Kong.

The data collected were entered into a personal computer and processed and analyzed by staff from the POP. The Statistical Package for the Social Sciences version 17.0 (SPSS Inc., Chicago, Illinois, USA) was used for all data analysis. Descriptive frequencies and means and standard deviations (SDs) were reported as appropriate. T- test and Chi-square test were used to compare the age, education, dental visit behavior frequency of food and beverage intake, caries experience according to the presence of signs and symptoms that can be related to dental erosion. The level of statistical significance was set at 0.05 level.

## Results

A total of 520 respondents (186 male and 334 female) completed the telephone survey (response rate, 75%; sampling error, ± 4.4%). Participants had a mean age of 37.1 ± 6.0 (Table 2). They were most commonly aged 41 to 45 years (41%), with smaller proportions aged 36 to 40 years (24%), 25-30 years (21%), and 31 to 35 years (14%). Most participants had completed only up to secondary school education (64%). Furthermore, 30% had completed tertiary education or above, and 6% had attained education at only primary level or below.

**Table 2:** Age, meals per day taken, gender, education, dental visit behaviour and caries experience according to risk to dental erosion of the respondents.

		Risk to dental erosion		
	All	Yes*	No	p-value
Age (n = 514)	37.1 ± 6.0	36.5 ± 6.3	37.9 ± 5.5	0.008
				
No. of meals/day (n = 494)	5.4 ± 2.1	5.3 ± 2.0	5.5 ± 2.3	0.355
				
Gender (n = 520)				0.441
Male	36%	104 (37%)	82 (34%)	
Female	64%	175 (63%)	159 (66%)	
				
Education level (n = 517)				< 0.001
Primary or below	6%	13 (5%)	20 (9%)	
Secondary	64%	163 (58%)	167 (70%)	
Tertiary	30%	103 (37%)	51 (21%)	
				
Dental visit (n = 499)				0.212
At least 1/yr	22%	59 (22%)	52 (23%)	
Less than 1/yr	67%	176 (65%)	157 (69%)	
Never	11%	36 (13%)	19 (8%)	
				
Caries experience (n = 520)				0.086
Yes	79%	212 (76%)	198 (82%)	
No	21%	67 (24%)	43 (18%)	

* had at least 3 out of the 5 sign and symptoms asked

**Figure 1:**
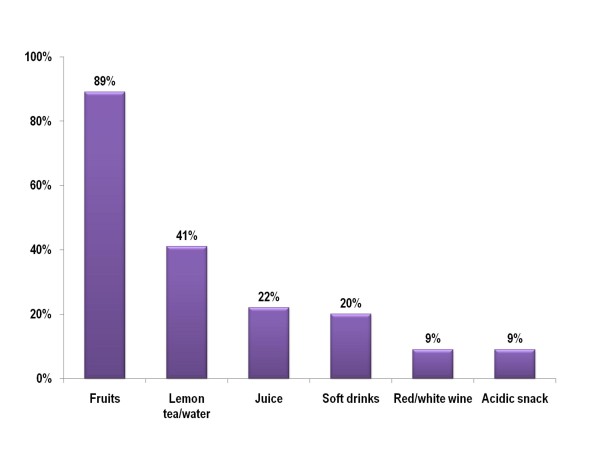
**Figure 1**. Dietary analysis

Questionnaire items on dental visits showed that 64% of respondents attended dental check-ups at least once a year, 21% visited a dentist less than once a year, and 11% had never visited a dentist. The majority (79%) had ever had caries. In the dietary analysis, fruit (89%) and lemon tea/water (41%) were the most commonly consumed food or drink (Figure 1). Respondents had a mean of 5.4 ± 2.1 meals per day (Table 2) and 36% had at least 6 meals per day. However, there is no statistically significant association between self-reported signs and symptoms that can be related to dental erosion and the number of meals, consumption of fruit, lemon tea/water, fruit juice, or soft drinks.

**Figure 2.**
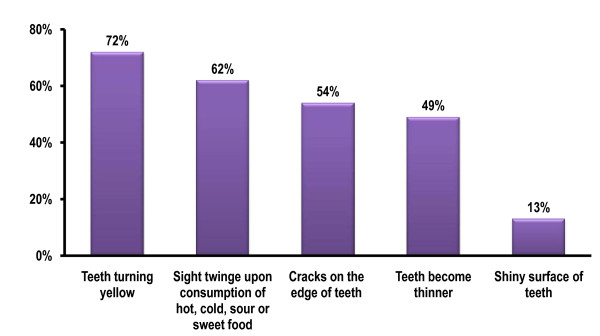
Figure 2.

When the respondents were asked if they had ever noticed or experienced signs and symptoms such as teeth becoming thin and turning yellow; a slight twinge when consuming hot, cold, sour, or sweet food; and cracks on the edge of their teeth or teeth with a shiny surface (Figure 2), most of them (92%) reported at least one of the above mentioned signs and symptoms which could be related to dental erosion. About half of the respondents (46%) reported they had with at least 3 of the 5 sign and symptoms and they were regard as people at risk to dental erosion (Table 2). They were younger and better educated than those who did not experience many sign and symptoms. Almost half of the respondents (48%) answered that they had noticed these signs and symptoms for the past 6 or more years, whereas 37% had noticed them for the past 2 to 5 years. However, 73% did not know what signs and symptoms of dental erosion were and 70% of them had never heard about dental erosion. In addition, more than half of the respondents (53%) could not differentiate between dental erosion and dental caries.

When respondents were actually told about dental erosion, 62% said they would seek professional dental treatment and care. In addition, 59% of respondents said that brushing their teeth immediately after meals would help to prevent dental erosion.

## Discussion

This study is a telephone survey to study the dietary habits and knowledge about dental erosion. Nearly all people in Hong Kong can have access to a telephone as there were, on average, 99 telephone lines per 100 households and penetration rate of mobile telephone was over 150% in 2008 [17]. Despite the popularity of telephones in Hong Kong, conducting telephone survey has limitations such as people's telecommunication practices. Unfortunately, it is difficult to gauge how much impact this limitation had on the results of this survey. Nevertheless, these telecommunication changes can be expected to cause increasing challenges for researchers who use telephone as a means to communicate with their study subjects. Recent changes in telecommunication regulations such as the development of do-not-call registries may also make it more difficult for researchers to contact some people via telephone. Another limitation is that some people especially those in the low socio-economic class, may have limited access to telephones.

Despite questions were asked on signs and symptoms that can be related to dental erosion, this study did not attempt to measure the prevalence of dental erosion. A clinical examination is necessary to confirm the presence of dental erosion. The diagnosis of dental erosion in reality is not straightforward since tooth erosion often involves various additional, modifying factors [2]. Dental erosion often occurs with abrasion and attrition; hence a collective term of "non-caries tooth loss" is often used instead. Even when the etiology of dental erosion is established, the subjectivity of diagnosis by evaluators can affect the assessment outcome. In addition to the difficulties in assessing the prevalence and severity of dental erosion, and other manifestations of tooth wear, there is also currently no standard method of measurement [18]. The indices proposed so far are often unreliable for use in longitudinal assessments, particularly for the extent of enamel loss and close monitoring of the progression of tooth wear. The most recent development is a proposal by a group of experts who formulated a new scoring system called Basic Erosive Wear Examination, or the BEWE index [19].

Dental erosion has been a condition of little interest to clinicians and researchers for many years. In addition, it is often not recognized and can be confused with other forms of tooth wear such as abrasion and attrition, which frequently coexist with erosion. This situation has changed during the past few years among dentists [20]. However, it is not known if the general public is aware of the importance of acid erosion of teeth. This survey of young to middle-aged people in Hong Kong found that although about half of the respondents had noticed signs and symptoms that can be related to dental erosion, the majority had never heard about dental erosion before, and more than half even mixed it up with dental caries. In addition, they were not aware of its symptoms and consequences. This lack of public knowledge about dental erosion has also been reported in the UK [21]. Dental health promotion and education are thus very important to help the public understand dental erosion and its damaging effects.

This study found many Chinese Hong Kong adults have frequent meals and frequently consume fruit, lemon tea/water, fruit juice, and soft drinks. However, this study could not find a significant association of symptoms that can be related to dental erosion and number of meals, consumption of fruit, lemon tea/water, fruit juice or soft drinks. Some researchers also have not found a relationship between dental erosion and fruit and acidic drinks [22,23]. Nevertheless, others have found a significant association [5,24,25]. Despite its high mineral content, enamel can be eroded if it has prolonged or frequent contact with acid.

All carbonated drinks, including soda (even diet varieties) contain a lot of acid such as citric, phosphoric and carbonic acids which can rapidly dissolve enamel on teeth. Energy drinks such as Gatorade had pH values of around 2.9 and they also contained a substantial amount of fermentable sugars. Owens [26] concluded that these energy drinks had strong erosive potential on teeth possibly due to a high buffering capacity. Large-sized drinks (710 ml) are popular in many restaurants and fast-food shops, and more damage is done when people drink large amounts and hold the drink in the mouth for some time before swallowing. Permanent teeth of adolescents are more prone to acid attack by the soft drinks because their teeth have immature enamel [26]. In addition, the high titratable acidity or the strong buffering capacity of the soft drinks can resist pH changes brought by the salivary actions and precipitate a prolonged period of oral acidity [27].

This survey found most of Hong Kong Chinese adults (89%) frequently took fruit. Fruit is considered a healthy food among Chinese people. Pure fruit juice is often advocated as a healthy drink, but it actually contains a lot of acid and can have a very low pH. Moreover, its high buffering capacities may induce a prolonged drop in oral pH that can contribute to dental erosion. Orange juice contains citric acid and its acidity (pH is 3.45) is comparable to that of soft drinks [27]. Alcoholic fruit-flavored drinks (alcopops) are also commonly drunk by young adults and have been implicated as an etiological factor in dental erosion [28]. With changes in lifestyles, Mandel [29] observed the consumption of wine has increased. Wine derives its acidity mostly from its contained tartaric and malic acids and from smaller concentrations of citric and succinic acids [30].

Early signs and symptoms of dental erosion may not be noticeable. In this study, about three quarters of the respondents noticed their teeth were turning yellow. Many respondents in this study said they experienced a slight twinge when consuming hot, cold, sour or sweet food, thereby indicating that enamel had worn or eroded away, exposing dentin and making the teeth sensitive. Furthermore, about half of the respondents complained of cracks on the edges of teeth and thinning teeth. Even though these symptoms may also due to abrasion and attrition and that dental erosion often co-exists with abrasion and attrition, it is essential to conduct a clinical survey to assess the prevalence and severity of dental erosion among the Chinese people.

Dental erosion may progress without much notice and is difficult to treat in advanced stages. Therefore, to raise public awareness of dental erosion, dentists can recommend ways of preventing dental erosion when people attend dental check-ups. Suggestions could include reducing or eliminating intake of carbonated drinks; alternatives are water, milk, or tea or coffee without sugar. Bassiouny *et al*. [31] reported that black and green tea are minimally erosive and should be encouraged as regular beverages. The erosive effect of tea is similar to that of water, which has no erosion potential. Carbonated drinks, when taken, should be drunk quickly and a straw can be used to push the liquid to the back of the mouth. It is important not to swish them around or hold them in the mouth for long periods. Davies *et al*. [32] found that all the sour candies they tested were erosive and some were even more acidic than orange juice. Hence, sour candies should be avoided.

Dental erosion is not dental caries, yet many respondents in this study were confused and had a misconception that brushing immediately after consuming acidic food and beverage would prevent the damaging effects of dental erosion. In fact, the tooth surface when softened by acids from food and beverage is more vulnerable to damage by tooth brushing, especially when a person uses an abrasive toothpaste and has an improper or over-zealous brushing technique. After consuming acidic food or drinks, people can rinse with water to help neutralize the acids. Chewing sugar-free gum can produce more saliva to promote remineralisation [33]. It is desirable to wait an hour or so before brushing teeth, or else brushing immediately will increase damage to enamel and dentin [34]. Brushing should then use a soft toothbrush and any toothpaste containing fluoride [35]. Sensitivity may be reduced by brushing with fluoride toothpaste that is specially formulated (such as containing potassium nitrate). Tooth mousse contains a casein phosphopeptite and amorphous calcium phosphate can also be used to control dental erosion [36].

Finally, this study provides a good background to formulate further epidemiological studies and effective health promotion programs to prevent dental erosion among Chinese adults. The demographic data on age, educational level and occupation according to gender of the adult sample in this study are all in agreement with the report of the latest Hong Kong Population By-Census [37], and the response rate was satisfactory. Hence, the sampling method used in our telephone survey can be considered to be a reliable and useful for further studies of dental erosion. In addition, the prevalence of self-reported signs and symptoms of dental erosion may be used to estimate the sample size required for subsequent clinical evaluation and study on the prevalence of dental erosion among Chinese people in Hong Kong.

## Conclusion

Chinese adults living in Hong Kong frequently consume food and drink. Fruit and lemon tea/water are the most commonly consumed acidic food and beverage. The majority have noticed signs and symptoms that can be related to dental erosion. A clinical survey to assess dental erosion is thus necessary. Although most adults make regular dental visits, many are not aware of and have little knowledge about dental erosion. Dental health education is essential to help the public understand dental erosion and its damaging effects.

## Competing interests

The authors declare that they have no competing interests.

## Authors' contributions

The three authors contributed equally to this work, and they read and approved the final manuscript.

## Authors' informations

Dr CH Chu is an associate professor in the Faculty of Dentistry, Ms Karie KL Pang is Assistant Director of the Public Opinion Program, Social Science Research Center, and Dr Edward CM Lo is a professor in the Faculty of Dentistry, The University of Hong Kong.

## Pre-publication history

The pre-publication history for this paper can be accessed here:

http://www.biomedcentral.com/1472-6831/10/13/prepub

## Supplementary Material

Additional file 1 **Questionnaire on Dietary behavior and knowledge of dental erosion**. Questionnaire on Dietary behavior and knowledge of dental erosion. Click here for file
