# Supplementary material for: Dietary behavior and knowledge of dental erosion among Chinese adults
Source: BMC Oral Health. 2010 Jun 3;10:13. doi: 10.1186/1472-6831-10-13 (PMC2894740; doi:10.1186/1472-6831-10-13)
Supplement: Additional file 1 — Questionnaire on Dietary behavior and knowledge of dental erosion. Questionnaire on Dietary behavior and knowledge of dental erosion. [file 1472-6831-10-13-S1.PDF]

GREY
HEALTHCARE GROUP

【A Survey on Hong Kong People's Dietary Behavior in Relations to Acid Erosion】

Final Draft： February 25, 2008

Target Respondents: Hong Kong citizens aged 25 to 45 and having symptoms of acidic erosion of teeth. The symptoms of acidic erosion of teeth include teeth turning yellowish, having a shinier surface, having a thinner edge, mild fragmentation in edges and feeling sore or painful when drinking cold and hot beverages or eating sour and sweet food.

Interviewers:

Good evening, sir/madam, this is an interviewer from the Public Opinion Programme (POP) of the University of Hong Kong. We are conducting a survey on people's opinions on teeth health in Hong Kong. I would like to invite you to participate in an interview which will take only a few minutes. I would like to stress that all information you provide will be kept strictly confidential and used for aggregate analysis only. Is it okay for us to start this survey?

The target of this survey is Hong Kong citizens aged 25 to 45.

Do you belong to this group? (If not, interviewers can ask 'Are there any other eligible target respondents in your household. Can I speak to him/her?' If there are none, interiew ends and say thank you for their co-opeartion.)

1. Are you aged 25 to 45?

- ☐ Yes
- ☐ No (interview ends)
- ☐ Refuse to answer (interview ends)

2. Compared to when you were a teenager, do your teeth now: (Read out all answers)

	Yes	No
1. become more yellowish in colour?		
2. become more shiny and smooth?		
3. have thinner edges?		
4. have fractured edge?		
5. feel sore or painful when drinking cold/ hot beverage and eating sour/ sweet food?		

2008 Grey Healthcare's Property. Copyrights Reserved. Confidential.

☐ Yes, for any one of the above answers → Interview continues
☐ None of the above cases (interview ends)
☐ Refuse to answer (interview ends)

I. People's perception towards acidic erosion of teeth

1. **Have you ever suffered from dental caries, including teeth which had undergone cavity filling?**
   ☐ Yes
   ☐ No
   ☐ Refuse to answer

2. **Have you ever heard of "acidic erosion of teeth"?**
   ☐ Yes
   ☐ No
   ☐ Don't know/ Hard to say
   ☐ Refuse to answer

3. **Do you think acidic erosion of teeth is equivalent to dental caries?**
   ☐ Yes
   ☐ No
   ☐ Don't know/ Hard to say
   ☐ Refuse to answer

4. **Prior to this interview, do you know what the symptoms of acidic erosion of teeth are?（Interviewers not to read our options, multiple answers allowed）**
   ☐ Don't know
   ☐ Teeth turning yellowish
   ☐ Teeth having a smoother and shinier surface
   ☐ Teeth having a thinner edge
   ☐ Teeth having a fractured edge
   ☐ Feeling sore or painful when drinking cold and hot beverages or eating sour and sweet food
   ☐ Suffering from dental caries
   ☐ Others:___________
   ☐ Refuse to answer

<u>To be read out by interviewer:</u>
The symptoms of acidic erosion of teeth include teeth turning yellowish, having a shinier and smoother surface, having a thinner edge, mild fragmentation in edges and feeling sore or painful when drinking cold and hot beverages or eating sour and sweet food.

2008 Grey Healthcare's Property. Copyrights Reserved. Confidential.

II. People's attitude towards acidic erosion of teeth

5. **At about what age have you first had symptoms of acidic erosion of teeth?**
   - ☐ 20 years old or earlier
   - ☐ 21-30 years old
   - ☐ 31-40 years old
   - ☐ 41-45 years old
   - ☐ Cannot remember/ don't know / hard to say （Skip to Q8）
   - ☐ Refuse to answer （Skip to Q8）

6. **In other words, how long have you suffered from these symptoms？(Half year to be counted as 1 year, e.g. 1 and a half year = 2 years, 3 and a half years = 4 years)**
   - ☐ 1 year or less
   - ☐ 2-3 years
   - ☐ 4-5 years
   - ☐ 6-10 years
   - ☐ 11-20 years
   - ☐ More than 20years
   - ☐ Cannot remember/ don't know / hard to say
   - ☐ Refuse to answer

III. Impacts of symptoms of acidic erosion of teeth on peopls's daily lives

7. **Are you worried about your teeth having the following symptoms? (Interviewers to read out answers, multiple answers allowed)**
   - ☐ Teeth turning yellowish
   - ☐ Teeth having a smoother and shinier surface
   - ☐ Teeth having a thinner edge
   - ☐ Teeth having a fractured edge
   - ☐ Teeth become smaller due to acidic erosion
   - ☐ Not worried at all/ None of the above
   - ☐ Don't know / hard to say
   - ☐ Refuse to answer

IV. How people deal with symptoms of acidic erosion of teeth

8. **When symptoms of acidic erosion of teeth appear, what would you usually do about it？（Interviewers not to read our options, multiple answers allowed）**
   - ☐ Do nothing
   - ☐ Brush your teeth immediately after ingestion
   - ☐ Brush your teeth more frequently
   - ☐ Brush your teeth harder
   - ☐ Chew sugarless gum after ingestion
   - ☐ Chew food more gently
   - ☐ Change toothpaste of different brands

2008 Grey Healthcare's Property. Copyrights Reserved. Confidential.

- ☐ Use dental floss, have scaling or bleaching
- ☐ Drink beverages and eat food that is too cold or hot less frequently
- ☐ Eat sweet food less frequently
- ☐ Eat sour food less frequently
- ☐ Drink beverages and eat food that is too irritating less frequently
- ☐ Consult a dentist
- ☐ Have a dental check-up regularly
- ☐ Others:___________
- ☐ Don't know / hard to say
- ☐ Refuse to answer

9. **Do you think the following are correct treatments for acidic erosion of teeth? (Interviewers to read out answers, order to be randomized by computer, multiple answers allowed)**
   - ☐ Brush your teeth immediately after ingestion
   - ☐ Brush your teeth more frequently
   - ☐ Brush your teeth harder
   - ☐ Use lemon slices to rub against and bleach teeth which have turned yellowish
   - ☐ None of the above
   - ☐ Don't know / hard to say
   - ☐ Refuse to answer

V. Relationship between people's eating habits and acidic erosion of teeth

10. **Do you usually have: (Interviewers to read out answers)**
    - ☐ Breakfast
    - ☐ Lunch
    - ☐ Dinner
    - ☐ None of the above
    - ☐ Don't know / hard to say
    - ☐ Refuse to answer

11. **In a day, how often on average do you drink beverages other than pure water and pure tea, and eat in between your regular meals (including soft drinks, coffee, snacks, fruit, dessert etc)? Please include the frequency in between your breakfast and lunch, lunch and dinner, and after dinner until your go to bed. Count it as once even if you have only consumed 1 piece of candy.**
    - ☐ None
    - ☐ 1-2 time(s)
    - ☐ 3-4 times
    - ☐ 5-6 times
    - ☐ 7-8 times
    - ☐ 9-10 times
    - ☐ More than 10 times
    - ☐ Don't know / hard to say
    - ☐ Refuse to answer

2008 Grey Healthcare's Property. Copyrights Reserved. Confidential.

12. **Lastly, do you consume the following beverage or food as a habit? (Interviewer to read out 1-6 accordingly. If respondent answers yes, probe for frequency)**

	Several times a day	Once a day	Several times a week	Once a week	Once in several weeks	No
1. Fruit						
2. Fruit juice						
3. Soft drinks						
4. Lemon tea/ water						
5. Red wine/ white wine						
6. Snack that are sour (e.g. sour candies, ginger, dried plum)						

☐ Others:___________
☐ Don't know / hard to say
☐ Refuse to answer

VI. Respondents' demographic information

I'd like to know some of your personal particulars in order to facilitate our analysis. Please be rest aasured that all information your provided will be kept strictly confidential.

13. Gender (Interviewer inputs directly)

☐ Male
☐ Female

14. Age (range)

☐ 25-30
☐ 31-35
☐ 36-40
☐ 41-45
☐ Refuse to answer

15. Education Attainment

☐ Primary or below
☐ Secondary
☐ Tertiary or above
☐ Refuse to answer

2008 Grey Healthcare's Property. Copyrights Reserved. Confidential.

16. Occupation

- ☐ Working full-time
- ☐ Working part-time
- ☐ Unemployed
- ☐ Student
- ☐ Retired
- ☐ Housewife
- ☐ Others:___________
- ☐ Refuse to answer

17. Personal monthly income

- ☐ Below HK$5,000
- ☐ HK$5,001-HK$10,000
- ☐ HK$10,001-HK$20,000
- ☐ HK$20,001-HK$30,000
- ☐ HK$30,001-HK$40,000
- ☐ HK$40,001-HK$50,000
- ☐ Above HK$50,000
- ☐ Refuse to answer

18. How often do you consult a dentist in a year on average?

- ☐ Less than once
- ☐ Once
- ☐ Twice
- ☐ 3 times
- ☐ 4 times
- ☐ 5 times
- ☐ More than 5 times
- ☐ Don't consult a dentist
- ☐ Don't know/hard to say
- ☐ Refuse to answer

Thank you for your time. If you have any questions regarding this interview, you can call XXXX-XXXX to talk to our supervisor Ms Louise Pun, or the Human Research Ethics Committee for Non-Clinical Faculties of the University of Hong Kong at XXXX-XXXX during office hours to verify this interview's authenticity and confirm my identity. Good-bye!

-END-

2008 Grey Healthcare's Property. Copyrights Reserved. Confidential.
